# Genetic diversity and geographic distribution of *Bemisia tabaci* (Gennadius) (Hemiptera: Aleyrodidae) genotypes associated with cassava in East Africa

**DOI:** 10.1002/ece3.379

**Published:** 2012-10-01

**Authors:** Habibu Mugerwa, Marie E C Rey, Titus Alicai, Elijah Ateka, Hellen Atuncha, Joseph Ndunguru, Peter Sseruwagi

**Affiliations:** 1 National Crops Resources Research InstituteP.O. Box 7084, Kampala, Uganda; 2 Department of Horticulture, Jomo Kenyatta University of Technology and AgricultureP.O. Box 62000-00200, Nairobi, Kenya; 3 Mikocheni Agricultural Research InstituteEastern Zone, P.O. Box 6226, Dar Es Salaam, Tanzania; 4 School of Molecular and Cell Biology, University of the WitwatersrandP.O. Box 2050, BraamFontein, Johannesburg, South Africa

**Keywords:** Cytochrome oxidase I (mtCOI), genetic diversity, geographic distribution, mitochondria, whitefly

## Abstract

The genetic variability of whitefly (*Bemisia tabaci*) species, the vectors of cassava mosaic begomoviruses (CMBs) in cassava growing areas of Kenya, Tanzania, and Uganda, was investigated through comparison of partial sequences of the mitochondria cytochrome oxidase I (mtCOI) DNA in 2010/11. Two distinct species were obtained including sub-Saharan Africa 1 (SSA1), comprising of two sub-clades (I and II), and a South West Indian Ocean Islands (SWIO) species. Among the SSA1, sub-clade I sequences shared a similarity of 97.8–99.7% with the published Uganda 1 genotypes, and diverged by 0.3–2.2%. A pairwise comparison of SSA1 sub-clade II sequences revealed a similarity of 97.2–99.5% with reference southern Africa genotypes, and diverged by 0.5–2.8%. The SSA1 sub-clade I whiteflies were widely distributed in East Africa (EA). In comparison, the SSA1 sub-clade II whiteflies were detected for the first time in the EA region, and occurred predominantly in the coast regions of Kenya, southern and coast Tanzania. They occurred in low abundance in the Lake Victoria Basin of Tanzania and were widespread in all four regions in Uganda. The SWIO species had a sequence similarity of 97.2–97.7% with the published Reunion sequence and diverged by 2.3–2.8%. The SWIO whiteflies occurred in coast Kenya only. The sub-Saharan Africa 2 whitefly species (Ug2) that was associated with the severe CMD pandemic in Uganda was not detected in our study.

## Introduction

*Bemisia tabaci* (Gennadius) (Hemiptera: Aleyrodidae) is widely distributed worldwide and is composed of a complex of more than 24 morphologically indistinguishable cryptic species (Dinsdale et al. 2010; De Barro et al. 2011; Shu-sheng et al. 2012). The 3.5% pairwise genetic divergence identified by Dinsdale et al. (2010) as being the boundary separating different species is further supported by evidence for either complete or partial mating isolation between a number of the putative *B. tabaci* “species” (Xu et al., 2010, Wang et al. 2011). The species level groups identified by Dinsdale et al. (2010) conform to the following 24 well-defined high-level phylogeographical groups (names of associated biotypes are placed in parentheses where applicable): Mediterranean (Q, J, L, Sub-Saharan Africa Silverleaf); Middle East-Asia Minor 1 (B, B2); Middle East-Asia Minor 2; Indian Ocean (MS); Asia I (H, M, NA); Australia/Indonesia; Australia (AN); China 1 (ZHJ3); China 2; Asia II 1 (K, P, ZHJ2); Asia II 2 (ZHJ1); Asia II 3; Asia II 4; Asia II 5 (G); Asia II 6; Asia II 7 (Cv); Asia II 8; Italy (T); Sub-Saharan Africa 1; Sub-Saharan Africa 2 (S); Sub-Saharan Africa 3; Sub-Saharan Africa 4; New World (A, C, D, F, Jatropha, N, R, Sida); and Uganda. *Bemisia tabaci* has gained increased importance as a crop pest and a vector of plant viruses, particularly geminiviruses in the genus *Begomovirus* (family, *Geminiviridae*) in tropical and subtropical regions of the world (Poulston and Anderson 1997). In sub-Saharan Africa, *B. tabaci* is a major vector of cassava mosaic begomoviruses (CMBs) and cassava brown streak viruses (CBSVs), the causative agents of cassava mosaic disease (CMD) and cassava brown streak disease (CBSD), respectively. The two diseases cause devastating yield losses in cassava (Maruthi et al. 2005; Legg et al. 2011).

An epidemic of severe CMD was first reported to cause devastating effects (100% yield loss) to cassava crops in Uganda in the 1990s (Otim-Nape et al. 1997). Subsequent studies associated the spread of the epidemic with unusually high whitefly populations (Legg 1999; Otim-Nape et al. 2000; Colvin et al. 2004). The high whitefly populations were believed to be a result of several factors including an invasive whitefly (Legg et al. 2002), whiteflies feeding on plants infected with the severe EACMV-UG2 (Colvin et al. 2004, 2006) and the widespread occurrence of whitefly-susceptible cassava varieties (Omongo et al. 2004, 2012) in the severe CMD pandemic affected areas.

Using the mitochondria cytochrome oxidase I (mtCOI) marker (Frohlich et al. 1999), Legg et al. (2002) identified two distinct cassava-associated *B. tabaci* genotype clusters, designated as Uganda 1 (Ug1) and Uganda 2 (Ug2), which currently fall in genetic groups designated as sub-Saharan Africa 1 (SSA1) and sub-Saharan Africa 2 (SSA2), respectively (Dinsdale et al. 2010). The Ug1 occurred in areas “ahead” of the epidemic front, whereas Ug2 was the predominant population at the “front”. It was suggested that the Ug1 was the indigenous or local population, whereas the Ug2 could be an “invader” population with its closest relatives from Cameroon in West Africa. The occurrence of the two *B. tabaci* species on cassava in Uganda was further confirmed by Maruthi et al. (2004), although with diminishing proportions of the Ug2 species in 2003 (Sseruwagi 2005).

Cassava mosaic disease continues to devastate cassava crops in East and Central Africa threatening the lives of over 200 million people (Legg et al. 2006). As a consequence, a number of programs have been instituted by African governments through the national agricultural research systems (NARS), and different local and international stakeholders to monitor the spread of the disease and enforce mitigation measures. However, limited research has been conducted to establish the current situation of the *B. tabaci* species associated with the disease. A clear understanding of whitefly species associated with the spread of the CMBs in the region would be invaluable to assist the development of durable integrated pest and disease management strategies.

This study sought to establish the genetic diversity and geographic distribution of *B. tabaci* associated with CMD and CBSD on cassava in East Africa (Kenya, Tanzania, and Uganda). We use the term “genotypes” or “haplotypes” to refer to genetically distinct sequences within the species boundary sharing <3.5% nucleotide similarity, whereas “species” refers to a genetic group of closely related sequences exhibiting more than 3.5% divergence with other species as described by Dinsdale et al. (2010).

## Materials and Methods

### Study area

The study was conducted in three East African countries: Kenya, Tanzania, and Uganda. In each country, major cassava producing areas were demarcated as follows: Western, Nyanza, and Coast provinces (Kenya); Lake Victoria Basin, Southern zone, and Coast zone (Tanzania); and central, northern, eastern, and western regions (Uganda).

*Kenya*: Western and Nyanza provinces share a similar agro-ecology, which is characterized by: bimodal rainfall ranging from 950 to 1500 mm annually, temperature ranges between 18.4 and 25.4°C, altitude of 900–1800 m, and a savannah grass land. The coast province has rainfall ranging from 500 to 1000 mm annually, temperature ranges between 22.4 and 30.3°C, altitude of 900–1800 m, and a savannah grass land (http://www.infonet-biovision.org/default/ct/690/agrozones).

*Tanzania*: Lake Victoria Basin is characterized by: bimodal rainfall ranging from 1000 to 2000 mm annually, temperature ranges between 17 and 28°C, altitude of 1000–1800 m, and has savannah vegetation with scattered tall trees. The Southern zone has unimodal rainfall ranging from 600 to 800 mm annually, temperature ranges between 18 and 28°C, altitude of 200–600 m and is composed of woodland, bush land thickets, and grassland. Coast zone has a bimodal rainfall ranging from 750 to 1200 mm annually, temperature ranges between 22 and 30°C, altitude of under 300 m, and a savannah grass land (http://www.fas.usda.gov/pecad/highlights/2005/09/tanzania_2005/images/TZ_AEZ.htm).

*Uganda*: The cassava growing regions are characterized by: temperatures ranging from 25 to 31°C, and altitude ranging between 900 and 1500 mm. Central region has bimodal rainfall averaging 1000 mm annually and vegetation is savannah grassland with moderate biomass. Northern region has both unimodal and bimodal rainfall averaging 800 mm annually and covered with short savannah grassland. Eastern region has a bimodal rainfall ranging from 750 to 1200 mm annually with short savannah grassland. The western region has bimodal rainfall ranging from 1000 to 1500 mm annually and is a forest savannah area. (http://www.fao.org/ag/AGP/AGPC/doc/Counprof/uganda/uganda.htm).

### Whitefly collection

**Figure 1:**
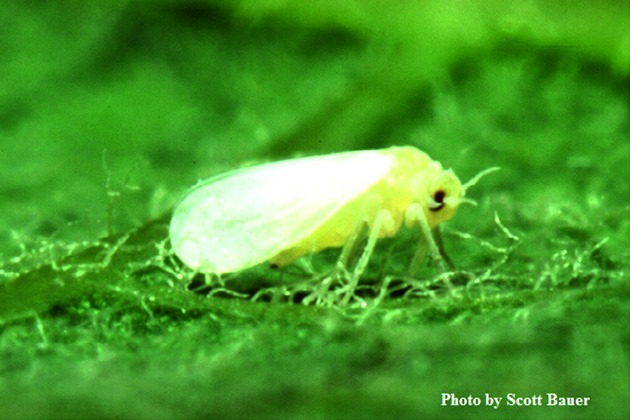
Adult whitefly (*Bemisia tabaci*)

Adult whiteflies (Fig. 1) were collected using an aspirator from 3- to 5-month-old cassava plants from different regions in Kenya, Tanzania, and Uganda (Table 1) and stored in 70% ethanol. Geo-coordinates (latitude and longitude) were recorded using a Geographical Positioning System (GPS) for each sampled location.

**Table 1:** Whitefly mitochondria cytochrome oxidase I sequences used in the study

Genotype name	Name in database	Country	Region/province	GenBank accession no.
Ke-Nyz23a	KeNyanza23a	Kenya	Nyanza	JQ286408
Ke-Nyz23b	KeNyanza23b	Kenya	Nyanza	JQ286409
Ke-C1	KeCoastC1	Kenya	Coast	JQ286410
Ke-C2	KeCoastC2	Kenya	Coast	JQ286411
Ke-C3	KeCoastC3	Kenya	Coast	JQ286412
Ke-C4	KeCoastC4	Kenya	Coast	JQ286413
Ke-C5	KeCoastC5	Kenya	Coast	JQ286414
Ke-C6	KeCoastC6	Kenya	Coast	JQ286415
Ke-C7	KeCoastC7	Kenya	Coast	JQ286416
Ke-C8	KeCoastC8	Kenya	Coast	JQ286417
Ke-N1	KeNyanzaN1	Kenya	Nyanza	JQ286418
Ke-N2	KeNyanzaN2	Kenya	Nyanza	JQ286419
Ke-N3	KeNyanzaN3	Kenya	Nyanza	JQ286420
Ke-N4	KeNyanzaN4	Kenya	Nyanza	JQ286421
Ke-N5	KeNyanzaN5	Kenya	Nyanza	JQ286422
Ke-N6	KeNyanzaN6	Kenya	Nyanza	JQ286423
Ke-N7	KeNyanzaN7	Kenya	Nyanza	JQ286424
Ke-W1	KeWesternW1	Kenya	Western	JQ286425
Ke-W2	KeWesternW2	Kenya	Western	JQ286426
Ke-W3	KeWesternW3	Kenya	Western	JQ286427
Ke-W5	KeWesternW5	Kenya	Western	JQ286429
Ug-2a	UgArua2a	Uganda	Northern	JQ286430
Ug-2b	UgArua2a	Uganda	Northern	JQ286431
Ug-3a	UgSoroti3a	Uganda	Eastern	JQ286432
Ug-3b	UgSoroti3b	Uganda	Eastern	JQ286433
Ug-24a	UgKatakwi24a	Uganda	Eastern	JQ286434
Ug-43a	UgMasindi43a	Uganda	Western	JQ286435
Ug-43b	UgMasindi43b	Uganda	Western	JQ286436
Ug-43c	UgMasindi43c	Uganda	Western	JQ286437
Ug-43d	UgMasindi43d	Uganda	Western	JQ286438
Ug-43e	UgMasindi43e	Uganda	Western	JQ286439
Ug-43f	UgMasindi43f	Uganda	Western	JQ286440
Ug-92a	UgAdjumani92a	Uganda	Northern	JQ286441
Ug-101a	UgKitgum101a	Uganda	Northern	JQ286442
Ug-101b	UgKitgum101b	Uganda	Northern	JQ286443
Ug-113a	UgMpigi113a	Uganda	Central	JQ286444
Ug-113b	UgMpigi113b	Uganda	Central	JQ286445
Ug-157a	UgNakaseke113a	Uganda	Central	JQ286446
Tz-8b	TzBunazi8b	Tanzania	Kagera, Lake zone	JQ286447
Tz-10b	TzRugera10b	Tanzania	Kagera, Lake zone	JQ286448
Tz-16a	TzOukasole16a	Tanzania	Kagera, Lake zone	JQ286449
Tz-16b	TzOukasole16b	Tanzania	Kagera, Lake zone	JQ286450
Tz-18a	TzChamugisha18a	Tanzania	Kagera, Lake zone	JQ286451
Tz-23a	TzNyakanzi23a	Tanzania	Kagera, Lake zone	JQ286452
Tz-23b	TzNyakanzi23b	Tanzania	Kagera, Lake zone	JQ286453
Tz-28a	TzMpomvu28a	Tanzania	Mwanza, Lake zone	JQ286454
Tz-49a	TzKwasunga49a	Tanzania	Tanga, Coastal zone	JQ286455
Tz-53a	TzMabukweni53a	Tanzania	Tanga, Coastal zone	JQ286456
Tz-57a	TzMkata57a	Tanzania	Tanga, Coastal zone	JQ286457
Tz-57b	TzMkata57b	Tanzania	Tanga, Coastal zone	JQ286458
Tz-58a	TzMbwewe58a	Tanzania	Tanga, Coastal zone	JQ286459
Tz-58b	TzMbwewe58b	Tanzania	Tanga, Coastal zone	JQ286460
Tz-62a	TzMbinga62a	Tanzania	Ruvuma, Southern zone	JQ286461
Tz-62b	TzMbinga62b	Tanzania	Ruvuma, Southern zone	JQ286462
Tz-65a	TzMkinga65a	Tanzania	Ruvuma, Southern zone	JQ286463
Tz-68a	TzMkoha68a	Tanzania	Ruvuma, Southern zone	JQ286464
Tz-70a	TzMatomondo70a	Tanzania	Ruvuma, Southern zone	JQ286465
Tz-70b	TzMatomondo70b	Tanzania	Ruvuma, Southern zone	JQ286466
Tz-75a	TzLikalangilo75a	Tanzania	Tanga, Coastal zone	JQ286467
Tz-77a	TzRwengu77a	Tanzania	Ruvuma, Southern zone	JQ286468
Tz-81a	TzMajala81a	Tanzania	Ruvuma, Southern zone	JQ286469
Tz-83a	TzMasaiyaleo83a	Tanzania	Mtwara, Southern zone	JQ286470
Tz-90a	TzMasaiyaleo90a	Tanzania	Mtwara, Southern zone	JQ286471
Tz-90b	TzMasaiyaleo90b	Tanzania	Mtwara, Southern zone	JQ286472
Tz-91a	TzTikule91a	Tanzania	Lindi, Southern zone	JQ286473
Tz-93a	TzWangurukuru93a	Tanzania	Lindi, Southern zone	JQ286474
Tz-93b	TzWangurukuru93b	Tanzania	Lindi, Southern zone	JQ286475
Tz-95b	TzIkwiriri95b	Tanzania	Coast, Coastal zone	JQ286476
Tz-97a	TzNgunja97a	Tanzania	Coast, Coastal zone	JQ286477
Tz-H1	TzMwanzaH1	Tanzania	Mwanza, Lake zone	JQ286478
Tz-H4	TzTangaH4	Tanzania	Tanga, Coastal zone	JQ286479
Tz-H9	TzKageraH9	Tanzania	Kagera, Lake zone	JQ286480
Tz-H12	TzKageraH12	Tanzania	Kagera, Lake zone	JQ286481
Tz-H15	TzKageraH15	Tanzania	Kagera, Lake zone	JQ286482
Tz-H18	TzMwanzaH18	Tanzania	Mwanza, Lake zone	JQ286483
Tz-H20	TzMwanzaH20	Tanzania	Mwanza, Lake zone	JQ286484
Tz-H24	TzTangaH24	Tanzania	Tanga, Coastal zone	JQ286485
Tz-H27	TzMwanzaH27	Tanzania	Mwanza, Lake zone	JQ286486
Tz-H30	TzMwanzaH30	Tanzania	Mwanza, Lake zone	JQ286487

### Extraction of whitefly DNA

Three adult female whiteflies were randomly selected from each collection site. Each individual insect was ground in 10 μL of lysis buffer (5 mM Tris-HCl, Ph8.0, 0.5 mM EDTA, 0.5% Nonidet P-40, 1 mg/mL proteinase K) using the tips of 0.2 μL polymerase chain reaction (PCR) tubes. The lysis product was incubated for 15 min at 65°C and further 10 min at 95°C. Subsequently, it was centrifuged (∼60 sec) briefly and placed immediately on ice prior to PCR amplification. Lysis was carried out as described by Frohlich et al. (1999).

### PCR amplification of mtCOI DNA and sequencing

A total of 79 (Kenya – 21, Tanzania – 41, and Uganda – 17) whiteflies were used to study the genetic variability and distribution of cassava-associated *B. tabaci* genotypes in cassava growing areas of Kenya, Tanzania, and Uganda in 2010/11. Amplification of mitochondria cytochrome oxidase I (mtCOI) DNA was achieved by the use of a primer pair MT10/C1-J-2195 (5′-TTGATTTTTTGGTCATCCAGAAGT-3′) and MT12/L2-N-3014 (5′-TCCAATGCACTAATCTGCCATATTA) as per Simon et al., 1994. A DNA template of 5 μL was used in a PCR reaction mixture of 25 μL, containing 1× *Taq* buffer with Mg^2+^, 0.2 mM deoxynucleotide triphosphate (dNTPs), 0.32 mM each of primers MT10 and MT12, and 0.625U *Taq* DNA polymerase.

Initial denaturation of template DNA was conducted for 3 min followed by 30 cycles of denaturation at 94°C for 30 sec, primer annealing at 52°C for 30 sec, and extension at 72°C for 1 min. The final extension of 10 min was run at 72°C and the reaction held at 4°C in a Perkin Elmer DNA thermal cycler. Electrophoresis of PCR products was run in 1% agarose gel stained in ethidium bromide in 1× TAE buffer in a submarine gel unit and visualized using ultraviolet light. PCR products of the expected 850 bp size were obtained. Bands were excised from the agarose gel and purified for DNA cloning using a Qiagen gel Purification kit (QIAGEN, Venlo, the Netherlands) as per the manufacturer's procedure. Purified PCR products were cloned using the pGEM-T easy vector as per the manufacturer's instructions and sent to Bioscience Centre for Eastern and Central Africa, Nairobi for sequencing.

### Phylogenetic analysis of mtCOI sequence

Whitefly mtCOI sequences were edited manually to produce a consensus sequence of 817 bp for each individual whitefly using the Editseq program of DNAStar computer package (DNASTAR, Madison, Wisconsin). The edited sequences were aligned together with reference whitefly sequences obtained in the GenBank using Cluster W (weighted) (Thompson et al. 1994) algorithm option available in the MEGA 5.02 program (Tamura et al. 2011).

Aligned sequences were trimmed to about 650 bp and subjected to a heuristic search and subtree-pruning-regrafting branch swapping using maximum parsimony method available in MEGA 5.02. The ML tree was reconstructed using maximum parsimony optimality criterion with among-site rate variation corresponding with gamma distribution and a general-time – reversible substitution model with the rate matrix set to 1. For parsimony analysis, bootstrapping (Felsenstein 1985) was performed with PAUP using the heuristic option for 1000 replication at a 70% confident limit (Swofford 1998).

The following reference mtCOI sequences and their genbank accession numbers (indicated in brackets) were used for the analysis: Asia1 Thailand [AF164671]; Asia II China [AJ784261]; Asia II China [AJ783706]; Asia II China [AY686083]; Asia II China [AY686088]; Asia II China [AF418666]; Asia II 9 China Hunan [HM137313]; Asia II 10 China Guangdong [HM137356]; Asia II India [AJ748374]; Asia II Pakistan [AJ510065]; Asia III Taiwan [DQ174528]; Australia Bundaberg [GU086328]; Australia Indonesia [AB248263]; China [AY686085]; China [AY686091]; China 3 Yunnan [EU192050]; Italy [AY827596]; MedAmAF Pakistan [AJ510075]; MedAmAf BioB Reunion [AJ550177]; Med Syria [AB297897]; Ms Reunion [AJ550178]; New World Colombia [AJ550168]; SubsahAf1 Ug [AY057185]; SubSahAf1 Ug[AY057181]; SubsahAf1 Moz [AF344278]; SubsahAf1 SA [AF344264]; SubsahAf2 Ug [AY057194]; SubsahAF3 Cameroon [AF344257]; SubsahAf4 Cameroon [AF344247]; Uganda [AF418665]. The species used in the analysis as out group were *B. afer* [GU220055] and *B. subdecipens* [GU220056] (Dinsdale et al. 2010).

## Results

### Phylogenetic analysis of whitefly mtCOI sequences

**Figure 2:**
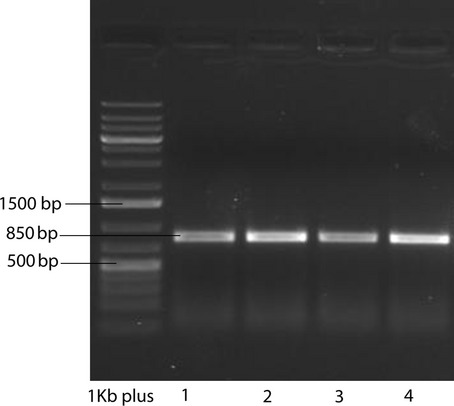
Agarose gel of PCR-amplified products of the expected 850 bp size. Lanes 1, 2, 3, and 4 are individual whitefly (*Bemisia tabaci*) insects.

A PCR fragment of the *mtCOI* gene (∼850 bp) was obtained for each adult whitefly using the primer pair: MT10/C1-J-2195 and MT12/L2-N-3014 (Fig. 2). A consensus sequence was obtained for each mtCOI 850 bp nucleotide sequence for the 79 whiteflies. The sequences have been deposited in the GenBank database as accession numbers JQ286408 to JQ286487 (Table 1).

**Figure 3:**
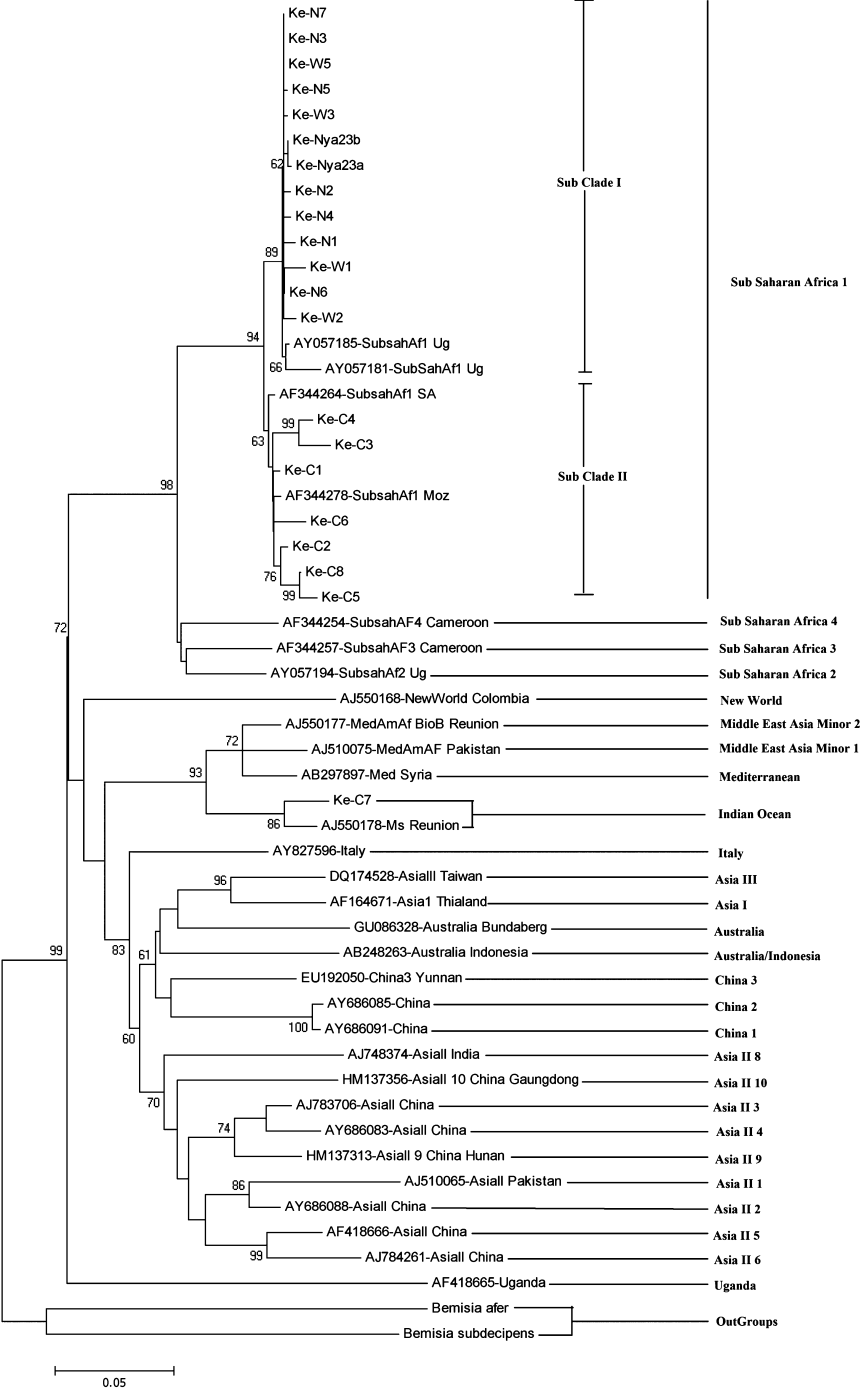
Phylogenetic tree based on the mitochondrial cytochrome oxidase I sequence for *Bemisia tabaci* collected in Kenya generated using maximum likilihood available in MEGA5 software program. *Bemisia subdecipens* and *B. afer* are included as outgroups. Whiteflies used in the study are labeled as follows: Ke-N1, Ke-N2, Ke-N3, Ke-N4, Ke-N5, Ke-N6, Ke-N7, Ke-Nyz23a,KeNyz23b (Nyanza region); Ke-W1, Ke-W2, Ke-W3, Ke-W5 (Western region); Ke-C1, Ke-C2, Ke-C3, Ke-C4, Ke-C5, Ke-C6, Ke-C7, Ke-C8 (Coastal region).

Based on the phylogenetic analysis of reference mtCOI sequences, members of *B. tabaci* generally group into distinct species cluster in the New World or Old World (Dinsdale et al. 2010). The New World *B. tabaci* from Colombia is genetically distinct from the Old World members from Australia, Mediterranean/North Africa/Middle East, Southeast Asia/Far East/India, and sub-Saharan Africa. Cassava-associated *B. tabaci* genotypes from Kenya grouped into the New World sub-Saharan Africa 1 (SSA-1) genetic clade with the exception of one genotype, which clustered with the SWIO genetic group (Delatte et al. 2011). Within the SSA-1 genetic group, the Kenyan genotypes clustered further in two sub-clades, hereafter named sub-clade I and sub-clade II. Sub-clade I contained Kenyan genotypes with a sequence similarity of 97.9–99.7% with the published Ug1 genotypes (Fig. 3) that occurred ahead of the severe CMD epidemic-affected areas in the 1990s (Legg et al. 2002). The sequences were 0.3–2.2% divergent (Table 2). Sub-clade II comprised of genotypes with 97.1–99.4% sequence similarity to the southern Africa (SA) genotypes from Mozambique and South Africa (Berry et al. 2004; Esterhuizen et al. 2012) (Fig. 3) and 0.6–2.9% sequence divergence (Table 2). Only one sequence clustered with the SWIO genetic group (Fig. 3) with 97.2–97.7% sequence similarity and 2.3–2.8% divergence (Table 2).

**Table 2:** A pairwise comparison of the mitochondrial cytochrome oxidase I (mtCOI) nucleotide sequence (representatives), expressed as percent nucleotide divergence between adult *Bemisia tabaci* populations identified on cassava in East Africa (Kenya, Tanzania, and Uganda) as calculated using Clustal algorithm (Thompson et al. 1994) Africa (2010/2011)

	**Genotype**	**1**	**2**	**3**	**4**	**5**	**6**	**7**	**8**	**9**	**10**	**11**	**12**	**13**	**14**	**15**	**16**	**17**	**18**	**19**	**20**	**21**	**22**	**23**	**24**	**25**	**26**	**27**
**1**	Ke-W2	-	0.8	0.5	3.3	2.0	3.7	19.3	0.9	0.6	0.5	2.0	1.7	1.5	0.6	1.1	2.0	1.9	2.5	2.5	2.2	0.8	1.5	2.0	20.3	20.9	9.5	24.6
**2**	Ke-N2		-	0.3	3.1	1.9	3.9	19.1	0.8	0.5	0.3	1.9	1.5	1.4	0.5	0.9	1.9	1.7	2.3	2.3	2.0	0.6	1.4	1.8	20.1	20.7	9.3	24.2
**3**	Ke-N3			-	2.8	1.5	3.6	18.7	0.5	0.2	0.0	1.5	1.2	1.1	0.2	0.6	1.5	1.4	2.0	2.0	1.7	0.3	1.1	1.5	19.7	20.3	9.0	24.0
**4**	Ke-C5				-	1.8	4.1	20.0	3.3	3.0	2.8	1.8	2.5	3.3	2.9	3.4	3.7	1.7	2.3	2.3	4.4	3.1	2.0	1.8	21.1	21.7	10.2	25.9
**5**	Ke-C1					-	2.8	18.5	2.0	1.7	1.5	0.6	1.2	2.0	1.7	2.2	2.5	0.5	1.1	1.1	3.1	1.9	0.8	0.6	19.5	20.1	9.0	24.7
**6**	Ke-C3						-	20.3	4.1	3.7	3.6	2.8	3.4	4.2	3.8	4.2	4.6	2.6	2.9	3.3	5.2	3.9	2.9	2.8	21.3	21.9	11.1	26.4
**7**	Ke-C7							-	19.3	18.9	18.7	18.1	18.6	19.1	18.9	19.3	19.3	18.3	17.7	18.4	20.5	18.7	18.5	18.4	2.3	2.8	18.3	25.2
**8**	Tz-10b								-	0.6	0.5	2.0	1.7	1.5	0.6	1.1	2.0	1.9	2.5	2.5	2.2	0.8	1.5	2.0	20.3	20.9	9.5	24.4
**9**	Tz-H9									-	0.2	1.7	1.4	1.2	0.3	0.8	1.7	1.5	2.2	2.2	1.8	0.5	1.2	1.7	19.9	20.5	9.2	24.2
**10**	Tz-H4										-	1.5	1.2	1.1	0.2	0.6	1.5	1.4	2.0	2.0	1.7	0.3	1.1	1.5	19.7	20.3	9.0	24.0
**11**	Tz-58b											-	1.2	1.7	1.7	2.2	2.5	0.5	1.1	1.1	3.1	1.9	0.8	0.6	19.1	19.7	9.0	23.8
**12**	Tz-70b												-	1.7	1.4	1.8	2.5	1.1	1.7	1.7	2.8	1.5	1.1	1.2	19.7	20.3	8.8	24.9
**13**	Tz-65a													-	1.2	1.7	2.6	1.9	2.5	2.5	2.6	1.4	1.5	2.0	20.1	20.7	9.7	24.0
**14**	Tz-43a														-	0.5	1.7	1.5	2.2	2.2	1.8	0.5	1.2	1.7	19.9	20.5	9.2	24.2
**15**	Ug-43d															-	2.2	2.0	2.6	2.3	2.3	0.9	1.7	2.2	20.3	21.0	9.5	24.5
**16**	Ug-43e																-	2.3	3.0	2.6	3.0	1.9	2.3	2.5	20.3	20.9	9.9	24.9
**17**	Ug-113b																	-	0.9	0.9	3.0	1.7	0.6	0.5	19.3	19.9	8.8	24.5
**18**	Ug-92a																		-	1.2	3.6	2.3	1.2	1.1	18.9	19.5	9.2	24.5
**19**	Ug-43b																			-	3.4	2.3	1.2	1.1	19.4	20.1	9.1	24.6
**20**	Sub Saharan Africa 1 Ug																				-	1.7	2.6	3.1	21.5	22.2	10.2	26.0
**21**	Sub Saharan Africa 1 Ug																					-	1.4	1.9	19.7	20.3	8.6	24.5
**22**	Sub Saharan Africa 1 SA																						-	0.8	19.5	20.1	9.0	24.7
**23**	Sub Saharan Africa 1 Moz																							-	19.4	20.1	9.0	24.6
**24**	Ms Reunion																								-	0.8	19.1	24.0
**25**	Ms Reunion																									-	19.7	24.2
**26**	Sub Saharan Africa 2 Ug																										-	24.9
**27**	*Bemisia afer*																											-

**Figure 4:**
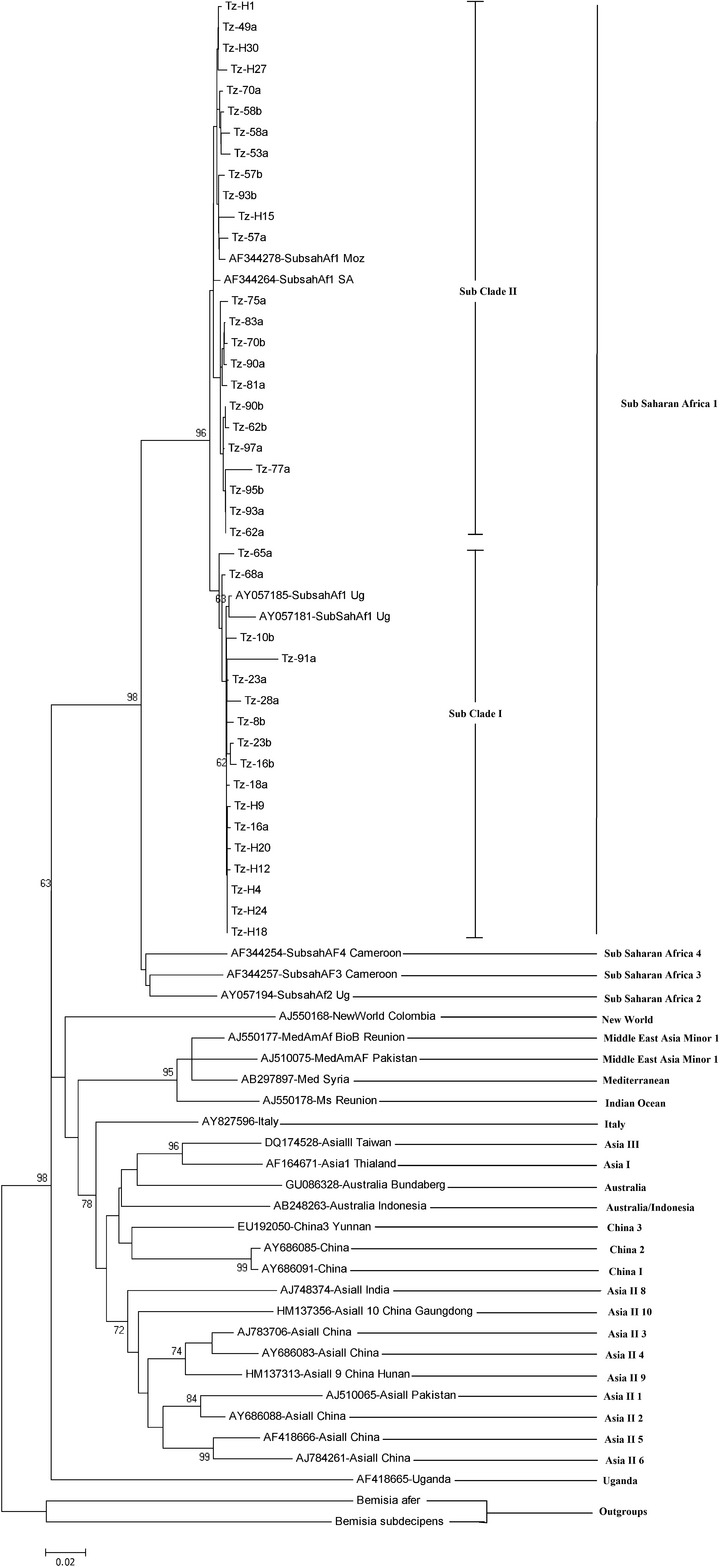
Phylogenetic tree based on the mitochondrial cytochrome oxidase I sequence for *Bemisia tabaci* collected in Tanzania generated using maximum likelihood available in MEGA5 software program. *Bemisia subdecipens* and *B. afer* are included as outgroups. Whiteflies used in the study are labeled as follows: Tz8b, Tz10a, Tz16a, Tz16b, Tz18a, Tz23a, Tz23b, Tz28b, TzH9, TzH12, TzH15, TzH18, TzH20, TzH27, TzH30 (Lake Victoria Basin), Tz49a, Tz53b, Tz57a,Tz57b, Tz58a, Tz58b, Tz75b, Tz95a, Tz95b, Tz97a, TzH4, TzH24 (Coastal region) and Tz62a, Tz62b, Tz65a, Tz68a, Tz70a, Tz70b, Tz75b, Tz77a, Tz81a, Tz83a, Tz90a, Tz91a, Tz93a, Tz93b (Southern zone).

Phylogenetic analysis of the mtCOI *B. tabaci* from Tanzania grouped all the sequences into the SSA-1 genetic group with two sub-clades as was the case for the Kenyan sequences (Fig. 4). A pairwise comparison of mtCOI sequences of the sub-clade I Tanzanian *B. tabaci* genotypes revealed a sequence similarity of 97.9–99.7% with reference to Ug1 genotypes, and a divergence of 0.3–2.2% among the sequences (Table 2). Sub-clade II genotypes shared 98–99.2% sequence similarity with SA genotypes (Fig. 4). The sequences diverged by 0.6–2% (Table 2).

**Figure 5:**
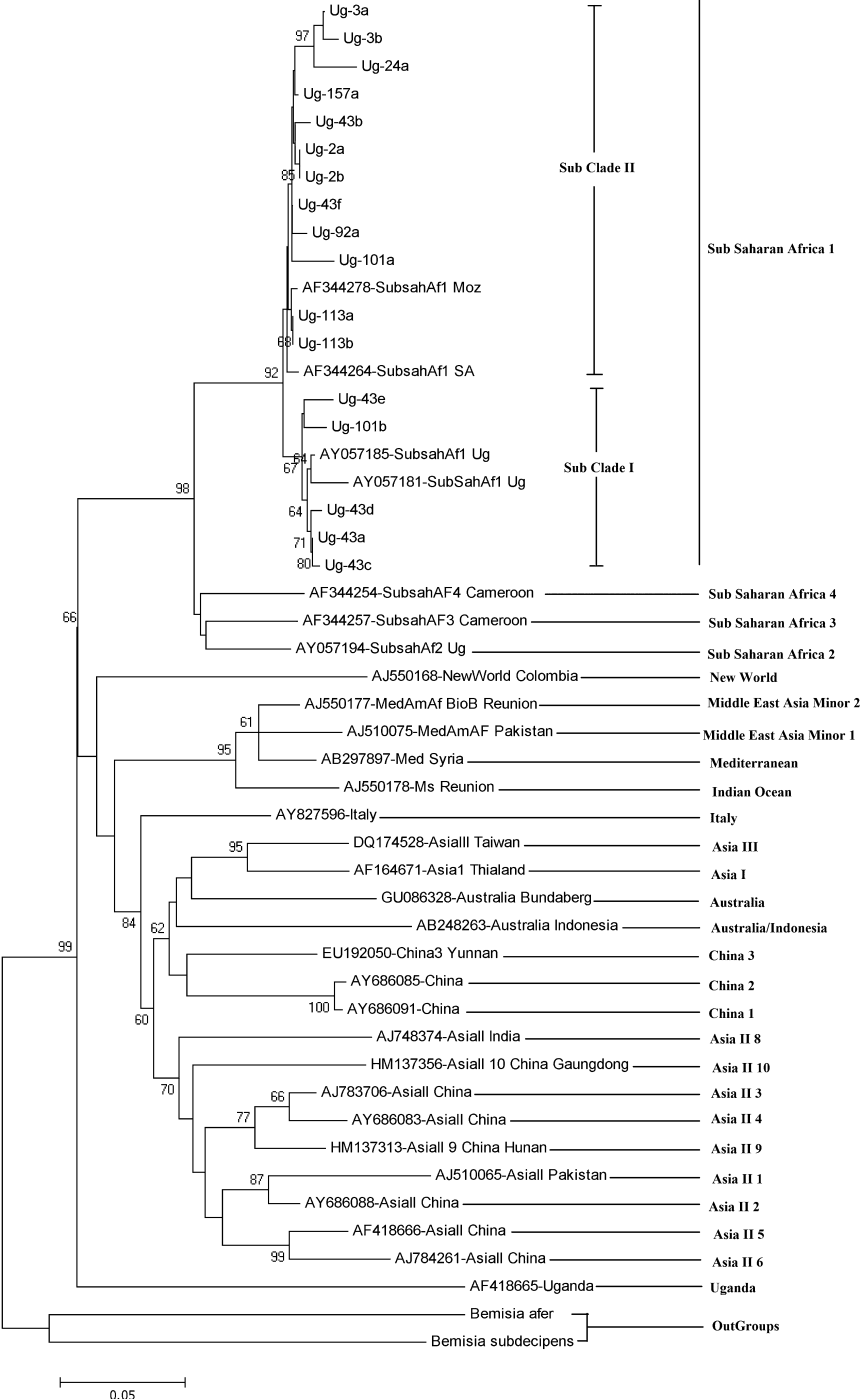
Phylogenetic tree based on the mitochondrial cytochrome oxidase I sequence for *Bemisia tabaci* collected in Uganda generated using maximum likelihood available in MEGA5 software program. *Bemisia subdecipens* and *B. afer* are included as outgroup. Samples are labeled as follows: Ug3a, Ug3b, Ug24a (East); Ug43a, Ug43b, Ug43c, Ug43d, Ug43e, Ug43f (West); Ug2a, Ug2b, Ug92a, Ug101a, Ug101b (North); Ug113a, Ug113b, Ug157a (Central) Uganda.

Similar to the Tanzanian grouping, results obtained for Ugandan *B. tabaci* grouped the genotypes into the SSA-1 genetic group with the sub-clades I and II (Fig. 5). Sub-clades I and II shared sequence similarities of 97.1–99.5% and 98.8–99.5% with Ug1 and SA genotypes, respectively. Within sub-clade I and II, the sequences diverged by 0.5–2.9% and 0.5–1.2%, respectively (Table 2

**Figure 6:**
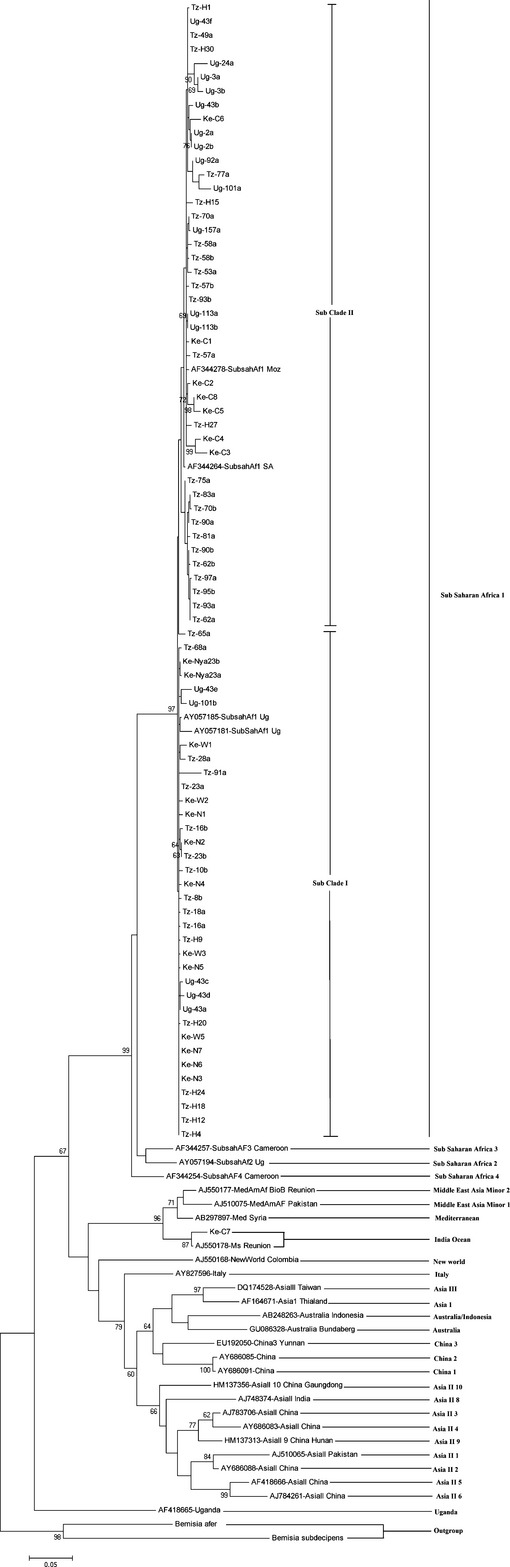
Phylogenetic tree based on the mitochondrial cytochrome oxidase I sequence for *Bemisia tabaci* collected in East Africa(Kenya, Tanzania, and Uganda) generated using maximum likelihood available in MEGA5 software program. *Bemisia subdecipens* and *B. afer* are included as outgroups

A combined phylogenetic analysis of mtCOI sequences of *B. tabaci* from all three countries (Kenya, Tanzania, and Uganda) grouped all the genotypes into SSA-1 genetic clade with exception of one genotype, which clustered with the SWIO genetic group (Fig. 6), confirming the results obtained for the individual countries. Sequences for *B. tabaci* in the two sub-clades (I and II) within SSA-1 genetic group were 0.3–2.8% divergent (Table 2). As expected, sequences of the Reunion *B. tabaci* from Kenyan, which grouped with SWIO genetic group diverged by 2.3–2.8% (Table 2).

### Analysis of molecular variance (ANOVA)

A hierarchical ANOVA (Excoffier et al. 2005) was conducted to assess the genetic differentiation of the *B. tabaci* populations in East Africa (Table 3). The two populations were grouped into the SSA-1 clade, with the two sub-clades (I and II), and SWIO with Reunion whitefly group. Comparative results from this study revealed significant differences among groups/clades (*P* < 0.001, FCT = 0.71529), among populations within groups (*P* < 0.001, FSC = 0.20534), and within populations (*P* = 0.008, FST = 0.77375). The highest contribution to the total variance was the differences among groups (71.53%). A similar result was obtained with the Tajima and Nei distance method (data not shown).

**Table 3:** Hierarchical analysis of molecular variance and *F*-statistics of genetic differentiation for East African *Bemisia tabaci* populations grouped according to species (groups), among populations within groups and within populations. The population structure was obtained using a pairwise difference distance method in ARLEQUIN version 3.1 (Excoffier et al. 2005), where Va is the variation due to differences among groups (Sub-clade I, Sub-clade II, and Reunion), Vb is the variation due to population differences within groups and Vc is the variation due to population differences

Source of variation	df	Sum of squares	Variance components	% of variation	*F*-statistics	*P*-value*
Among groups	1	98.812	40.95471 Va	71.53	0.71529	<0.0001
Among populations within group	1	141.357	4.34738 Vb	5.85	0.20534	<0.0001
Within populations	76	984.528	12.95432 Vc	22.63	0.77375	<0.0001
Total	78	1224.696	57.25641			

* *P* < 0.05.

### Geographic distribution of *B. tabaci* genotypes in East Africa

Results demonstrated a clear geographic distribution of two *B. tabaci* species belonging to the SSA-1 (sub-clade I and sub-clade II) and SWIO (Reunion) clades, in each country. The distribution within each region in the three countries was also evaluated. In Kenya, whiteflies were obtained in Western, Nyanza, and Coast provinces. The predominant species in all three provinces was the SSA-1, which comprised of 61.9% (13/21) sub-clade I (Ug1-like) and 33.3% (7/21) sub-clade II (SA-like) genotypes. The SWIO (Reunion) species comprised only 4.8% (1/21) (Table 4). Between regions, the sub-clade I genotypes were predominant in Nyanza (69.2%) and lowest in Western province (30.8%). Sub-clade I genotypes were conspicuously absent in the Coast province. Interestingly, the sub-clade II genotypes occurred in the Coast province only. The SWIO genotype occurred only in the Coast province (Table 4), whereas sub-clade I was only detected in Western and Nyanza provinces, and sub-clade II predominated in the Coast province.

**Table 4:** Geographic distribution of *Bemisia tabaci* species in Kenya, Tanzania, and Uganda, 2010/2011

		Species occurrence (%)			
			
		Sub-Saharan Africa 1			
				
Country	Region	Sub-clade I	Sub-clade II	South West Indian Ocean	No. of samples (*n*)
Kenya	Western	30.8(100)	0(0)	0(0)	4
	Nyanza	69.2(100)	0(0)	0(0)	9
	Coast	0(0)	100(87.5)	100(12.5)	8
		13	7	1	21
Tanzania	Lake zone	88.2(83.3)	12.5(16.7)	0(0)	18
	Coastal zone	0(0)	33.3(100)	0(0)	8
	Southern zone	11.8(13.3)	54.2(86.7)	0(0)	15
		17	24	0	41
Uganda	Western	80^1^(66.7)^2^	16.7(33.3)	0(0)	6
	Central	0(0)	33.3(100)	0(0)	3
	Eastern	0(0)	25.5(100)	0(0)	3
	Northern	20(25)	25.5(75)	0(20)	5
		5	12	0	17

1 Comparison across regions within each country.

2 Comparison between species/genotypes obtained within a region.

Tanzanian whiteflies were obtained in three zones, including Lake Victoria Basin, Coast, and Southern zones. The SSA-1 sub-clade I and II genotypes comprised 41.5% (17/41) and 58.5% (24/41) of the whiteflies from Tanzania, respectively (Table 4). Between region comparisons revealed that the sub-clade I (Ug1-like) genotypes were most abundant in the Lake Victoria Basin (88.2%). The sub-clade II (SA-like) genotypes were predominant in the Southern zone (54.2%). Sub-clade I dominated in the Lake Victoria Basin (83.3%), whereas the sub-clade II genotypes dominated in the Coast and Southern zones (Table 4).

In Uganda, whiteflies were obtained in four geographic regions: western, central, eastern, and northern regions. The SSA1 sub-clade II genotypes were the most abundant with 70.6% (12/17) occurrence (Table 4). Between regions, sub-clade I genotypes were most abundant in the western region (80%). Interestingly, no sub-clade I genotypes were detected in the central and eastern regions in this study. The sub-clade II genotypes occurred highest in central (33.3%) region and occurred in equal proportions in eastern and northern regions. Sub-clade I dominated in western region with 66.7% occurrence. On the other hand, sub-clade II was the dominant type in central, eastern, and northern regions (Table 4).

## Discussion

Using the *mtCOI* gene (Frohlich et al. 1999) as the molecular marker, our study reports the occurrence of two *B. tabaci* species belonging to two distinct clades/groups of whiteflies, namely sub-Saharan Africa 1 (SSA-1) and South West Indian Ocean Islands (SWIO), on cassava crops in Kenya, Tanzania, and Uganda. Phylogenetic trees were predicted using both maximum parsimony and the maximum likelihood methods with similar results, but only the maximum likelihood results are discussed. Genetic differentiation of the cassava-associated East African *B. tabaci* populations using ANOVA had the highest contribution to the total variance as differences among groups, which corroborates the results obtained with mtCOI sequence phylogenetic analysis. The SSA-1 species had two closely related sub-clades (I and II), which were earlier reported on cassava as Uganda 1 (Ug1) and southern Africa (SA) genotypes in Uganda (Legg et al. 2002) and southern Africa (Berry et al. 2004; Esterhuizen et al. 2012), respectively.

We reported here for the first time the occurrence of a Reunion whitefly species that clustered among the SWIO genetic group on cassava in Kenya. It is not clear whether this whitefly can reproduce on cassava as only the adults were used for the mtCOI analysis in this study. A Reunion (Ms) whitefly was also reported to colonize a number of non-cassava plants species, including *Commelina benghalensis*, *Gossypium hirsutum,* and *Phaseolus vulgaris* in Uganda (Sseruwagi et al. 2005). It is possible that the Reunion whitefly was just “visiting” or “feeding” at the time of sampling cassava. More definitive studies should be carried out to ascertain the colonization status of cassava by these whiteflies.

There was a clear geographic distribution of the cassava *B. tabaci* species in the East African region (Kenya, Tanzania, and Uganda). Previous studies reported the sub-clade I genotypes to occur widely in areas ahead of the severe CMD pandemic “front” indigenous populations, and were associated with very low numbers (Legg et al. 2002; Maruthi et al. 2004). However, in this study, the SSA-1 sub-clade I genotypes were not only widespread in Western and Nyanza provinces (Kenya), the Lake Victoria Basin and Southern zone (Tanzania), and western and northern regions (Uganda), but they also occurred in high population abundance (data not presented).

Previously, the super abundant whitefly populations were a characteristic of the severe CMD pandemic (Legg 1999; Otim-Nape et al. 2000; Colvin et al. 2004), which was attributed in part due to entry into Uganda of an invasive whitefly species of the sub-Saharan Africa 2 (SSA-2) genetic group with closest relatives in Cameroon, commonly referred to as the “invader/Ug2” (Legg et al. 2002). Interestingly, we did not detect the SSA-2 whitefly species on cassava in any of the three countries in this study. The diminishing occurrence of the SSA-2 whiteflies in the severe CMD-affected areas in Uganda was reported (Sseruwagi 2005). A likely explanation for their complete absence in this study and the resurgence of the SSA-1 (sub-clade 1) whitefly species in high populations in EA could be the result of backcrosses between the indigenous whitefly population (SSA-1 sub-clade I) and the SSA-2 invasive population, that resulted in a hybrid population with SSA-1 (sub-clade I) mtCOI and the invasive traits of the SSA-2 species (J. K. Brown, pers. comm.). Further studies are required to affirm the hybrid hypothesis.

On the other hand, the complete absence of the SSA-1 (sub-clade I) genotypes in Coast Kenya and Tanzania, and central and eastern Uganda could possibly be due to displacement by the SSA-1 (sub-clade II) genotypes, which comprised a majority of the whiteflies in these areas. Elsewhere, population increase in *B. tabaci*, particularly in areas where whiteflies were previously unimportant, was attributed to the appearance of new “biotypes”/species complexes (Bedford et al. 1994; Brown 2001). For example, in southwestern United States of America, the B-biotype/Middle East-Asia Minor species (De Barro et al. 2011) was introduced in the region through ornamental plants (Brown et al. 1995; Frohlich et al. 1999), where it increased steadily in distribution and abundance, ultimately displacing the “local” A-biotype/New World species (Costa et al. 1993; De Barro et al. 2011). On the other hand, the Middle East-Asia Minor species (B-biotype), which invaded southern Spain failed to displace the Q-biotype/Mediterranean indigenous species (Moya et al. 2001; De Barro et al. 2011). Displacement of indigenous *B. tabaci* species by the invasive Middle East-Asia Minor (B-biotype) and Mediterranean (Q-biotype) species has also been reported recently in China (Xu 2009; Chu et al. 2010; Crowder et al. 2010; Wang et al. 2011), and the Q-biotype has recently been reported in South Africa (SA) (Esterhuizen et al. 2012).

We report for the first time the occurrence of SSA-1 (sub-clade II) whitefly in East Africa. These whiteflies predominated in Coast Kenya, Southern and Coast Tanzania, and were low in the Lake Victoria Basin of Tanzania, and widespread in all four regions in Uganda. The SSA-1 (sub-clade II) may be the indigenous whitefly species in Coast Kenya, Southern and Coast Tanzania. It is generally believed to be the indigenous whitefly in southern Africa, including SA, Malawi, and Mozambique (Berry et al. 2004; Esterhuizen et al. 2012), countries that share common boundaries and climate with Tanzania. However, the occurrence of the SSA-1 (sub-clade II) whiteflies in the Lake Victoria Basin of Tanzania and in Uganda was unexpected and requires further investigation.

In conclusion, our results indicate that the two SSA-1 sub-clades (I and II), which group together as a single species in the SSA-1 *B. tabaci* genetic group due to their less than 3.5% divergence in the mtCOI (Dinsdale et al. 2010), could differ in important aspects of their biology, such as fecundity, virus transmission, and mating ability. This requires further investigation.
